# Small Epidemic Outbreak of Norovirus in the Pediatric Department of Brescia Civic Hospital (Northern Italy): Genomic Characterization and Phylogenetic Analysis

**DOI:** 10.1097/INF.0000000000005218

**Published:** 2026-03-18

**Authors:** Michele Pellegrino, Anna Bertelli, Laura Dotta, Sara Roversi, Stefania Marsico, Raffaele Badolato, Francesca Caccuri, Serena Messali

**Affiliations:** From the * Department of Pharmacy, Health and Nutritional Sciences, University of Calabria, Arcavacata di Rende, Italy; † Institute of Microbiology, ASST‐Spedali Civili of Brescia, Brescia, Italy; ‡ Department of Pediatrics, Department of Clinical and Experimental Sciences, ASST Spedali Civili of Brescia, University of Brescia, Brescia, Italy; § Section of Microbiology, Department of Molecular and Translational Medicine, University of Brescia, Brescia, Italy.

**Keywords:** human norovirus, nosocomial outbreak, phylogenetic analysis, pediatric patients, genetic analysis

## Abstract

**Background:**

Human noroviruses are a leading cause of acute gastroenteritis worldwide, with GII.4 Sydney 2012 [P16], being the predominant circulating genotype in Italy in recent years. Outbreaks in pediatric hospital wards represent a major public health concern.

**Methods:**

Eight stool samples collected between November 2023 and February 2024 at Brescia Civic Hospital were analyzed by real-time polymerase chain reaction, sequence-independent single-primer amplification and next-generation sequencing.

**Results:**

All isolates belonged to GII.4 Sydney 2012 [P16] genotype. Five isolates from December 2023 clustered tightly together, confirming a nosocomial outbreak with near-complete sequence identity. Isolates from November 2023, January 2024 and February 2024 were phylogenetically distinct, with 1 isolate (January 2024) clustering in a completely separated tree portion, carrying 30 amino acid substitutions and 237 synonymous mutations.

**Conclusions:**

Our analysis confirmed a nosocomial outbreak and underscored the substantial intra-genotypic diversity of GII.4 Sydney 2012 [P16] genotype. These findings highlight the importance of rapid diagnosis, infection control measures and continuous genomic surveillance to track the evolution and global circulation of epidemic noroviruses.

Human noroviruses (NVs) are the leading cause of acute nonbacterial gastroenteritis worldwide, affecting individuals of all ages but exerting a particularly high burden in young children, the elderly and immunocompromised patients.^1,2^ Transmission occurs primarily via the fecal–oral route, through person-to-person contact, contaminated food, water or environmental surfaces.^3^ Outbreaks are common in semi-closed settings such as hospitals, schools and nursing homes, where rapid viral spread is facilitated by a low infectious dose, environmental stability and prolonged viral shedding.^4,5^

NVs are positive-sense single-stranded RNA viruses belonging to the *Caliciviridae* family. Based on the sequence of the major capsid protein VP1, they are classified into 10 genogroups (GI–GX) and at least 49 genotypes, of which GI and GII infect humans more frequently than other genogroups.^6^ Over the last 2 decades, genogroup II, genotype 4 (GII.4) strains have been responsible for the majority of global outbreaks, owing to their ability to undergo rapid antigenic drift and recombination events, resulting in the emergence of novel variants with pandemic potential.^7,8^ Among these, the GII.4 Sydney variant, recognized in 2012, has constituted the predominant strain worldwide in recent years, often associated with the polymerase type P16 (GII.4 Sydney 2012 [P16]).^9–11^ This lineage has been linked to increased transmissibility and high attack rates in both community and healthcare settings.^12^

Nonetheless, in some European countries and the United States during 2023–2024 and 2024–2025 winter seasons, a significant increase in the circulation of GII.17 genotype over GII.4 was documented.^13,14^

Hospital outbreaks of NVs are particularly concerning in pediatric units, where children represent a susceptible population, and where viral spread can lead to significant morbidity, prolonged hospital stays and increased healthcare costs.^4,5^ Therefore, molecular epidemiology and genomic surveillance are essential not only to confirm outbreak occurrence but also to track strain circulation, monitor viral evolution and detect the emergence of new variants that may escape population immunity.^15,16^

In Italy, GII.4 Sydney 2012 [P16] has been repeatedly detected as the dominant circulating genotype in both community-associated and nosocomial outbreaks.^17,18^ In fact, previous works have already implemented deep sequencing methods to characterize local outbreaks.^19–21^

The present study describes the molecular and genomic characterization of 8 NV isolates obtained from stool samples collected in the pediatric department of Brescia Civic Hospital (Northern Italy) between November 2023 and February 2024. Five of these isolates were associated with an outbreak occurring in December 2023, while the remaining 3 were retrieved before and after the epidemic period. Through next-generation sequencing (NGS) and phylogenetic analysis, we aimed to clarify the genetic relationships among the isolates, confirm the outbreak and provide insights into NV strain circulation in Northern Italy. Our findings contribute to the understanding of intra-genotypic variability in GII.4 Sydney 2012 [P16] and underscore the importance of timely genomic surveillance for management, even in a small epidemic outbreak, and public health preparedness.

## MATERIALS AND METHODS

### Sample Collection and Preparation

Eight stool samples were collected from hospitalized patients at the pediatric department of Brescia Civic hospital (Northern Italy) from November 2023 to February 2024 and tested positive with real-time polymerase chain reaction (RT-PCR) Xpert Norovirus (Cepheid).

### Viral RNA Extraction

Viral RNA was automatically extracted from 200 µL of a filtered 10% stool suspension with the QIAamp DSP Virus Kit (Qiagen), according to the manufacturer’s instructions. Viral RNA was eluted in 30 µL of Buffer AVE and stored at −80°C until use.

### Sequence-Independent Single-Primer Amplification and Metagenomic NGS

First, RNA was reverse transcribed and amplified with the random priming method sequence-independent single-primer amplification (SISPA).^22^ Therefore, single-strand cDNA was obtained by incubating 40 pmol of Primer A (5′-GTTTCCCACTGGAGGATA-N9-3′) and SuperScript III RT Mix (2 μL 5× First-Strand Buffer, 1 μL H_2_O, 1 μL 12.5 mM dNTP mix, 0.5 μL 0.1 M DTT and 0.5 μL SS III RT) (ThermoFisher Scientific) at 42°C for 60 minutes. Second-strand DNA was synthetized by a 2-step addition of Sequenase DNA polymerase (ThermoFisher Scientific), as follows: 5 μL of Mix #1 (1 μL 5× Sequenase Buffer, 3.85 μL H_2_O, 0.15 μL Sequenase enzyme) was incubated for 8 minutes at 37°C, and then 0.6 μL of Sequenase Mix #2 (0.45 μL Buffer, 0.15 μL Sequenase) was added for a further 8 minutes to complete the second-strand synthesis. Primer A-labeled cDNA was then used for the Round B PCR reaction (94°C for 30 seconds, 50°C for 45 seconds, 72°C for 60 seconds, 40 cycles) while using AmpliTaq Gold polymerase (ThermoFisher Scientific) in the presence of 100 pmol Primer B (5′-GTTTCCCACTGGAGGATA-3′).

The PCR products were purified using 1.8× ratio AMPure XP beads (Beckman Coulter) to maximize the recovery of fragments above 200 bp in size and then eluted in 30 μL of DNase/RNase-free water. The purified products were quantified using the Qubit®DNA High-sensitivity Assay Kit (ThermoFisher Scientific). The Nextera DNA Flex Library Preparation kit (Illumina) was adopted to generate a multiplexed paired-end sequencing library. Briefly, 15 μL of the purified product was used in the tagmentation reaction to yield fragments >150 base pairs (bp), according to the protocol. The tagmented library underwent 8 cycles of PCR with indexed primers (IDT Technologies), followed by purification using AMPure XP beads (Beckman Coulter). The purified library was quantified with the Qubit Fluorometer (ThermoFisher Scientific) and loaded in a V2 300‐cycle sequencing cartridge to perform deep sequencing on the MiSeq platform (Illumina). The raw data were checked for quality using FastQC (https://www.bioinformatics.babraham.ac.uk). Subsequently, raw reads were trimmed (Trimmomatic ver. 0.39),^23^ removing any adaptor sequences, leading bases with PHRED <25 and trailing bases with PHRED <25, clipping the remainder of the read when a sliding window of 20 bases has an average PHRED < 25 (Q score > 25) and discarding reads with length <36 bases. Geneious software (v.11.1.5) (Biomatters Ltd) was implemented to analyze the filtered reads.

### NV3 Characterization and Genotyping

To characterize the NV3 isolate, through Bowtie2 in sensitive-local mode available in Geneious software, the trimmed reads were mapped on the most recent reference sequence of each of the GII genogroup 28 genotypes, available in the Human Calicivirus Genotyping Tool database.^24^ PP549880 is the accession number of the NV reference sequence on which the greatest number of the trimmed reads mapped. The obtained nearly full-length NV3 genomic sequence was characterized with Genome Detective ver 2.83,^25^ Nucleotide BLAST (NCBI) (https://blast.ncbi.nlm.nih.gov/Blast.cgi?PROGRAM=blastn&PAGE_TYPE=BlastSearch&LINK_LOC=blasthome), Norovirus Genotyping Tool^26^ and Human Calicivirus Genotyping Tool.^24^

### Amplicon-Targeted Approach of NGS

The molecular characterization of NV3 genomic sequences allowed the adoption of an amplicon-targeted approach of NGS to better characterize each NV isolate. Hence, on the basis of the alignment of 108 NV GII.4 Sydney 2012 [P16] whole-genome sequences sampled from 2015 until April 2024, available in Human Calicivirus Genotyping Tool database, 2 primers pairs and 1 reverse primer, to be coupled with UNP_47 forward primer described by Cotten and colleagues,^27^ were designed to reverse transcribe and amplify NV genome in the 3 overlapping portions (Table 1). Reverse transcription and amplification were performed with SuperScript IV One-Step RT-PCR (ThermoFisher Scientific) (25 µL of 2X Platinum SuperFi RT-PCR Master Mix; 0.5 µL of SuperScript IV RT Mix, 12 µL of the extracted viral RNA, the necessary microliters of nuclease-free H_2_O to reach a final volume of 50 µL; for FOR2_NV_BA, FOR3_NV_BA, REV3_NV_BA primers a final concentration of 0.75 µM was used, while for the other primers, a final concentration of 0.5 µM was chosen). The adopted thermal profile ran as follows: thermocycler pre-heating at 55°C; 55°C 10 minutes; 98°C 2 minutes; 40 cycles of 98°C 10 seconds, 10 seconds at the suitable annealing temperature according to the employed primer pair, 72°C 1 minute and 50 seconds; 72°C 5 minutes. PCR products were checked on 1.2% agarose gel. PCR products purification, quantification and NGS library preparation, NGS reads analysis, and genotyping of the NV isolates were performed as described in the previous paragraphs. Genomic sequence identity analysis was calculated in relation to NV1 with Geneious software (v.11.1.5) (Biomatters Ltd). The obtained genomic sequences were submitted to GenBank (https://www.ncbi.nlm.nih.gov/genbank/) with the following accession numbers: PX925253 (NV1), PX925254 (NV2), PX925255 (NV3), PX925256 (NV4), PX925257 (NV5), PX925258 (NV6), PX925259 (NV7), PX925260 (NV8).

**TABLE 1.** List of the Employed Primers for Reverse Transcription and Amplification of the Genome of NV Isolates in 3 Overlapping Amplicons

Amplicon	Direction	Primers	Sequence 5’–3’	Genomic Position (bp)	Tm (°C)
1	Forward	UNP_47	GTGAATGAAGATGGCGTCTAAC	1	55.5
1	Reverse	REV1_NV_BA	CAATGGCGAGCTCCTCATAGTAC	2808	60.9
2	Forward	FOR2_NV_BA	ATCTTCTAAGGAYATCAAGGAGG	2605	57.1
2	Reverse	REV2_NV_BA	CCATAGTATTTCACCTGGAGC	5315	55.8
3	Forward	FOR3_NV_BA	CATAACTGAACTCAARGAAGGTG	4957	55.5–57.1
3	Reverse	REV3_NV_BA	TCACATTACRCCCGTGACTCC	7518	59.7–62.4

The genomic position represents the first base where the primer 5’-end binds.

bp indicates base pair; Tm, melting temperature.

### Phylogenetic Analysis

kSNP3.0 program was employed to build a maximum parsimony phylogenetic tree of the 8 NV isolates’ genomic sequences plus the 108 GII.4 Sydney 2012 [P16] whole-genome sequences, already used for the primers design^28^ (Table, Supplemental Digital Content 1, https://links.lww.com/INF/G584).

## RESULTS

### Clinical Manifestation of NV-Positive Patients

The cohort consisted of 8 patients (5 males and 3 females) with a mean age of 6 years (range, 4 months–16 years). In November 2023, the first patient (NV8) was sampled during hospital admission for psychiatric exacerbations (Table 2); in February 2024, the NV6 patient was sampled on the same day she was admitted to the hospital because of a seizure during acute gastroenteritis that manifested with vomiting and diarrhea without fever 3 days before. In contrast, the other patients included in the present study developed gastrointestinal symptoms and were, therefore, tested for enteric infection a median of 4.5 days after hospital admission (range, 2–9 days). These patients were all admitted from the emergency department to the department of general pediatrics for various reasons, including respiratory syncytial virus bronchiolitis in the 4-month-old infant (NV1), hyperglycemia with polyuria and polydipsia in 2 adolescents with new-onset type 1 diabetes mellitus (NV2, NV4), acute respiratory distress due to *Pseudomonas aeruginosa* pneumonia in a fragile 8-year-old patient with type 1A ceroid lipofuscinosis (NV3), vomiting and fever at the onset of hemolytic anemia in a 15-month-old infant (NV5), acute respiratory symptoms in a 2-year-old boy with both Influenza A and Bocavirus infections (NV7). In all these patients, NV infection manifested as diarrhea without vomiting or fever, without any evidence of bloody diarrhea. At the time diarrhea occurred, mean C-reactive protein (CRP) levels were not elevated (mean value 3.7 mg/L; normal range <5 mg/L; range 1–24.3 mg/L). Only 2 patients (NV1 and NV5) showed neutrophilic leukocytosis (respectively, white blood cell count: 18,500 and 13,280 cell/µL) associated with a slight increase in CRP, whereas all the others had normal leukocyte counts. Overall, the mean white blood cell count was 9838 ± 5286 cells/µL, with a neutrophil count of 4130 ± 2790 cells/µL, a monocyte count of 948 ± 835 cells/µL and a lymphocyte count of 4570 ± 2418 cells/µL. None of the patients had evidence of immunodeficiency. Enteric symptoms lasted a maximum of 2 days and were not associated with any other complications. The 3 patients with concomitant respiratory infection (NV1, NV3 and NV7), including the fragile patient (NV3), received intravenous fluids, whereas the others (NV2, NV4, NV5, NV6, NV8) were treated with oral rehydration solution. None of the patients required prolonged hospitalization because of NV infection, and all were discharged once the main pathologic condition, which had led to hospital admission, had resolved. The retrospective data analysis did not allow to evaluate environmental factors such as common exposure outside the hospital among the included patients, nor their contacts.

**TABLE 2.** Clinical Characteristics of the Study Participants

Patient Designation	Patient Age at Admission	Admission Source	Date of Hospitalization	Hospital Ward	Date of Illness Onset	Symptoms	Date of Sample Collection	Date of Symptom Resolution	Date of Discharge	Underlying Disease
NV1	4 months	Emergency Department	10 Dec 2023	General Pediatrics	12 Dec 2023	Diarrhea	12 Dec 2023	14 Dec 2023	15 Dec 2023	RSV bronchiolitis
NV2	13 years	Emergency Department	13 Dec 2023	General Pediatrics	16 Dec 2023	Diarrhea	16 Dec 2023	18 Dec 2023	21 Dec 2023	DKA in type 1 diabetes at onset
NV3	8 years	Emergency Department	14 Dec 2023	General Pediatrics	16 Dec 2023	Diarrhea	17 Dec 2023	19 Dec 2023	29 Dec 2023	Pseudomonas pneumonia in encephalopathy
NV4	15 years	Emergency Department	14 Dec 2023	General Pediatrics	23 Dec 2023	Diarrhea	23 Dec 2023	24 Dec 2023	24 Dec 2023	Type 1 diabetes at onset
NV5	1 years	Emergency Department	20 Dec 2023	General Pediatrics	25 Dec 2023	Diarrhea	25 Dec 2023	27 Dec 2023	29 Dec 2023	Hemolytic anemia
NV6	1 years	Emergency Department	5 Feb 2024	Child and Adolescence Neuropsychiatry	2 Feb 2024	Vomiting and diarrhea; seizure	05 Feb 2024	06 Feb 2024	07 Feb 2024	None
NV7	2 years	Emergency Department	13 Jan 2024	General Pediatrics	15 Jan 2024	Diarrhea	15 Jan 2024	16 Jan 2024	16 Jan 2024	A Influenza
NV8	16 years	Emergency Department	25 Oct 2023	Child and Adolescence Neuropsychiatry	13 Nov 2023	Diarrhea	13 Nov 2023	15 Nov 2023	22 Nov 2023	Psychiatric disorder

### NV Detection and Genotyping

All 8 stool samples, collected from pediatric patients between November 2023 and February 2024 at Brescia Civic Hospital, tested positive for NV with the Xpert Norovirus RT-PCR assay. Subsequent amplification and sequencing allowed for genomic characterization of each isolate. First, the random priming method SISPA coupled with metagenomics NGS allowed the coverage of 75.6% of the NV3 genome and its characterization as belonging to GII.4 Sydney 2012 [P16] genotype. Explicitly, the VP1 gene was assigned to the GII.4 Sydney 2012 variant, while RNA-dependent RNA polymerase (RdRp) region corresponded to P16 type. This finding enabled the adoption of an amplicon-targeted NGS approach for sequencing the entire NV3 and the other NV isolate genomes. Therefore, 6 whole-genome sequences were successfully obtained for NV1, NV2, NV3, NV4, NV5 and NV8, and 2 nearly full-length genome sequences were obtained for NV6 and NV7. Therefore, to improve genome coverage of these 2 latter SISPA, followed by NGS, was performed. Overall, we were able to extend genome coverage to 89.7% for NV6 and to 74.2% for NV7 isolate. Overall, the genomic characterization confirmed that all these isolates belonged to GII.4 Sydney 2012 [P16] (Table 3).

**TABLE 3.** Molecular Analysis of NV GII.4 Sydney2012 [P16] Lineage Isolates Collected at Brescia Civic Hospital (November 2023–February 2024)

Isolate	Collection Date	Belonging to the Outbreak	Identity With Outbreak Cluster	Identity of *VP1* Gene	VP1 Gene Mutations	Identity of RdRp Gene	*RdRp* Gene Mutations
NV1 (reference)	12 Dec 2023	Yes	100%	100%	None	100%	None
NV2	16 Dec 2023	Yes	100%	100%	None	100%	None
NV3	17 Dec 2023	Yes	99.98%	99.97%	1 Silent Mutation	100%	None
NV4	23 Dec 2023	Yes	99.98%	99.97%	1 Silent Mutation	100%	None
NV5	25 Dec 2023	Yes	99.98%	99.97%	1 Silent Mutation	100%	None
NV6	13 Nov 2023	No	99.3%	99.60%	4 Silent Mutations, 4 Missense Mutations	99.3%	2 Silent Mutations, 1 Missense Mutation
NV7	15 Jan 2024	No	95.1%	99.00%	3 Silent Mutations, 2 Missense Mutations	94.7%	73 Silent Mutations, 7 Missense Mutations
NV8	5 Feb 2024	No	99.6%	99.00%	10 Silent Mutations, 2 Missense Mutations	99.6%	10 Silent Mutations, 0 Missense Mutation

RdRp indicates RNA-dependent RNA polymerase.

### Phylogenetic and Sequence Identity Analysis Confirm the Outbreak

**FIGURE 1.**
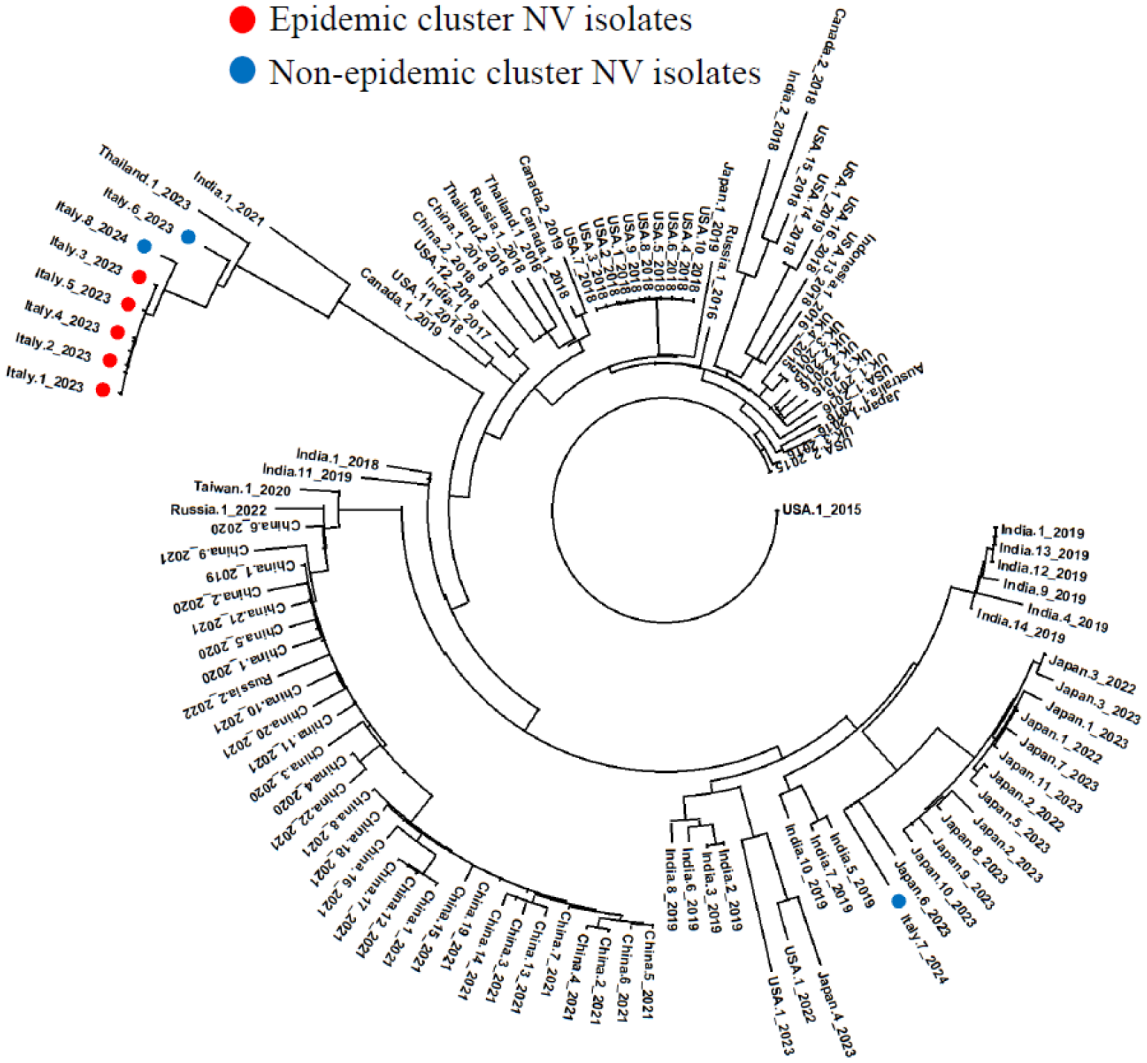
Phylogenetic relationship among GII.4 Sydney 2012 [P16] NV isolates. The parsimony tree includes 8 NV isolates collected at Brescia Civic Hospital from November 2023 to February 2024, plus 108 GII.4 Sydney 2012 [P16] NV isolates retrieved from the Human Calicivirus Genotyping Tool database from 2015 to April 2024. The NV isolates sampled in Brescia in December 2023 are highlighted in red dots, while blue dots depict the Brescia NV isolates gathered before and after the epidemic period. NV indicates norovirus.

To accurately determine evolutionary relationships among the 8 NV isolates within the GII.4 Sydney 2012 [P16] genotype, a parsimony tree was employed (Fig. 1). The dataset utilized in this analysis consists of 108 GII.4 Sydney 2012 [P16] genomic sequences retrieved from 2015 until April 2024 available in the Human Calicivirus Genotyping tool (Table, Supplemental Digital Content 1, https://links.lww.com/INF/G584). As shown in Figure 1, the 5 isolates collected in December 2023 (NV1–NV5) formed a tight cluster separated from all the other sequences, showed near-complete sequence identity (~100%), and shared a common ancestor. This confirmed the presence of a small nosocomial outbreak in the pediatric department during December 2023.

In contrast, the isolates collected before and after the epidemic period exhibited greater phylogenetic distance (Fig. 1 and Table 3). Specifically:

- **NV6 (November 2023**) shared 99.3% sequence identity with the outbreak cluster but was located on a separate branch, suggesting circulation of closely related but distinct strains before the outbreak. This was the index patient who was sampled during her hospitalization.
- **NV7 (January 2024**) showed marked divergence from all other study isolates (95.1% sequence identity), clustering in a completely different section of the phylogenetic tree, indicating significant genomic variation and possibly independent introduction into the hospital setting.
- **NV8 (February 2024**) represented the phylogenetically closest nonepidemic isolate to the outbreak isolates, sharing 99.6% sequence identity and a common ancestor with the outbreak cluster, but occupied a distinct phylogenetic branch. Among the NV isolates collected before and after the epidemic, a whole-genome sequence was obtained only for NV8 isolate, highlighting its phylogenetic correlation and sequence similarity with the outbreak cluster. This patient was sampled the day she was admitted to the hospital.

Particularly, sequence identity analysis focused on VP1 and RdRp nucleotide regions, because they are crucial for genotype assignment, with the P2 domain of VP1 representing the major antigenic site and displaying the highest variability rate. However, only partial data are available for VP1, because VP1 nucleotide sequences of isolates NV6 and NV7 were incomplete. As listed in Table 3, for VP1, only 1 single nucleotide polymorphism (SNP) leading to a synonymous mutation was observed for the outbreak isolates (NV3, NV4, NV5), while 12 SNPs, of which 83% represented synonymous substitutions, were counted for NV8, the phylogenetically closest nonepidemic isolate. Concerning RdRp sequence, no SNPs were found among the cluster isolates, while the nonepidemic samples carried from 3 to 80 SNPs, with NV7 exhibiting the highest substitution rate, with 80 SNPs, of which 91.3% synonymous (Table 3). These findings are in accordance with the distribution of Brescia isolates in the parsimony tree.

Despite being collected in a limited geographical area (Brescia province) and within a short 4-month time frame, the isolates demonstrated considerable intra-genotypic variability within the GII.4 Sydney 2012 [P16] lineage. The outbreak-related cluster showed a strong phylogenetic relationship, while sporadic isolates collected before and after the epidemic highlighted the dynamic evolution of the circulating strains.

## DISCUSSION

This study describes the molecular and genomic characterization of 8 NV isolates collected from the pediatric department of Brescia Civic Hospital during and around an outbreak of acute gastroenteritis that occurred in December 2023. Through whole-genome sequencing and phylogenetic analysis, we confirmed that the outbreak was caused by the GII.4 Sydney 2012 [P16] NV genotype, which has represented the globally predominant NV lineage for more than a decade after its first detection in 2012. Importantly, our findings highlight the remarkable intra-genotypic variability of this lineage, even within a restricted geographic area and a short time frame.

Clinical, epidemiologic and phylogenetic data suggested NV nosocomial transmission within the pediatric department during December 2023. In fact, none of the 5 pediatric patients who tested positive for NV in December 2023 had been admitted for gastroenteritis; they were hospitalized for other clinical conditions and were all tested for NV for the first time upon admission, on the same day they developed diarrhea. This finding provides further evidence of hospital-acquired NV infection, leading to a nosocomial outbreak in December 2023. Moreover, the 5 isolates collected in December 2023 clustered tightly and separately from all the other NV isolates, showing nearly 100% sequence identity and a shared common ancestor. These findings are in agreement with previous reports of GII.4 Sydney 2012 [P16] genotype being responsible for nosocomial outbreaks in both Europe and Asia, where its enhanced transmissibility and high viral loads have been associated with rapid person-to-person spread in hospital ward.^3,10,15^ The confirmation of this outbreak through phylogenetic analysis underscores the value of molecular epidemiology in distinguishing outbreak-related cases from coincidental sporadic infections, which may occur simultaneously in the same setting.

The expected level of genomic variability among epidemiologically linked strains deserves further discussion. Defining how many nucleotide changes can be tolerated before 2 strains should be considered unrelated is critical for the interpretation of whole-genome sequencing data in outbreak investigations. Prior studies of NV outbreaks based on the P2 domain have shown that nucleotide diversity within outbreaks is significantly lower than between unrelated strains, and have proposed empirical thresholds: for instance, SNP rates under 0.20%–0.50% have been considered as relaxed and rigorous cutoffs, respectively, to distinguish related strains in outbreak settings of NV using partial genome sequences.^29^ Moreover, Tao et al. specifically define that the SNP threshold for NV transmission clusters of approximately 2 SNPs (equivalent to ~0.33%–0.46% in the P2 region) could effectively discriminate linked from unlinked samples.^29^ In our study, 3 (NV3, NV4, and NV5) of the 5 epidemiologically linked strains differed from the cluster isolates NV1 and NV2 by only a single nucleotide change across the whole genome, whereas nonlinked strains showed substantially higher divergence, with a minimum of 114 SNPs. This marked separation provides a strong genomic signal supporting epidemiologic linkage in our dataset. Nonetheless, integrating these empirical SNP threshold frameworks from the literature^30^ strengthens the interpretation that low SNP counts can be indicative of direct transmission events, while higher divergence likely reflects distinct introductions or unrelated chains of transmission.

The 3 isolates collected outside the outbreak period revealed greater genomic heterogeneity. NV6, collected in November 2023, shared 99.3% identity with the outbreak cluster but was in a distinct phylogenetic branch, suggesting the circulation of a related strain before the outbreak. Furthermore, NV8 shared a common ancestor with the outbreak cluster yet was located on a separate branch, raising the possibility of a closely related variant.

Of particular interest was NV7 isolate, collected in January 2024, which displayed marked phylogenetic distance from the outbreak strains and clustered with Japanese sequences from 2023. Indeed, this isolate carried 7 nonsynonymous substitutions and 73 synonymous mutations just in the *RdRp* gene compared to NV1 *RdRp* sequence. Its phylogenetic proximity to Asian strains raises the possibility of international introduction, which has been described in other European settings where imported cases contributed to local NV diversity.^5,8^ These findings reinforce the notion that multiple lineages of GII.4 Sydney 2012 [P16] genotype may co-circulate within the same community, with distinct transmission chains overlapping in time and space.

The confirmation of an outbreak in a pediatric hospital unit underscores the clinical and epidemiologic relevance of NV in healthcare settings. Children represent a particularly vulnerable population, with NV infections associated with severe dehydration, prolonged hospital stays, and secondary household transmission.^31^ Rapid identification of outbreak-related strains, combined with prompt implementation of infection control measures such as patient isolation, strict hand hygiene, and environmental decontamination, remains essential to prevent nosocomial spread.^15,32^ Our findings emphasize the need for timely laboratory confirmation of suspected cases, given the nonspecific clinical presentation of viral gastroenteritis.

Nonetheless, our study presents some limitations due to the small number of analyzed samples and the short time of observation. Moreover, our research is restricted to the hospitalized children in a single center in Brescia, overlooking the NV strains circulating in the community and particularly, among adults. Interestingly, in Europe and in the United States during 2023–2024 and 2024–2025 winter seasons, among ≥65-year-old adults, an increased spread of NV GII.17 genotype over GII.4 was registered. Coherently with our data showing GII.4 Sydney 2012 [P16] circulation in children in 2023–2024 winter season, in foreign countries GII.4 Sydney 2012 [P16] has been predominant among children until February 2024, but subsequently GII.17 prevailed.^13,14^

In conclusion, our genomic analysis confirmed a small nosocomial outbreak of NV GII.4 Sydney 2012 [P16] genotype in the pediatric department of Brescia Civic Hospital in December 2023 and demonstrated the coexistence of genetically distinct intra-genotypic variants within a short time frame in Northern Italy. These findings highlight the dual challenges of controlling nosocomial spread and monitoring the ongoing evolution of NV. Rapid molecular diagnosis and strict infection control are critical to promptly mitigate the impact of NV outbreaks, while sustained genomic surveillance is crucial to detect the potential emergence of new pandemic strains.

## Supplementary Material


