# Supplementary material for: Small Epidemic Outbreak of Norovirus in the Pediatric Department of Brescia Civic Hospital (Northern Italy): Genomic Characterization and Phylogenetic Analysis
Source: Pediatr Infect Dis J. 2026 Mar 18;45(8):e281–7. doi: 10.1097/INF.0000000000005218 (PMC13340453; doi:10.1097/INF.0000000000005218)
Supplement: Supplementary file 1 [file inf-45-e281-s001.pdf]

SUPPLEMENTAL DIGITAL CONTENT 1. list of the GenBank accession numbers.

GenBank Accession Number	Phylogenetic Tree Designation
PX925253	Italy.1_2023
PX925254	Italy.2_2023
PX925255	Italy.3_2023
PX925256	Italy.3_2023
PX925257	Italy.5_2023
PX925258	Italy.6_2023
PX925259	Italy.7_2024
PX925260	Italy.8_2024
OR700742	USA.1_2023
OR700746	USA.1_2022
MT031821	USA.1_2019
MK752933	USA.16_2018
MK764016	USA.15_2018
MN897755	USA.14_2018
MN897756	USA.13_2018
MT029316	USA.12_2018
MT032004	USA.11_2018
MT238667	USA.10_2018
MT238668	USA.9_2018
MT238670	USA.8_2018
MT238671	USA.7_2018
MT238672	USA.6_2018
MT344180	USA.5_2018
MT537605	USA.4_2018
MT537633	USA.3_2018
MT537634	USA.2_2018
MT537941	USA.1_2018
KY947549	USA.1_2016
KX907727	USA.2_2015
KY947550	USA.1_2015
KY887599	UK.3_2016
KY887600	UK.2_2016
KY887601	UK.1_2016
KY887602	UK.4_2015
KY887603	UK.3_2015
KY887604	UK.2_2015
KY887605	UK.1_2015
PP549880	Thailand.1_2023
MW305708	Thailand.2_2018
MW305723	Thailand.1_2018
ON329737	Taiwan.1_2020
OP712198	Russia.2_2022
OP901694	Russia.1_2022
MK483909	Russia.1_2018
KY210980	Russia.1_2016
LC777250	Japan.11_2023
LC790060	Japan.10_2023
LC790061	Japan.9_2023
LC790063	Japan.8_2023
LC790064	Japan.7_2023
LC790065	Japan.6_2023
LC790066	Japan.5_2023

LC790067	Japan.4_2023
LC790068	Japan.3_2023
LC790069	Japan.2_2023
LC790070	Japan.1_2023
LC790057	Japan.3_2022
LC790058	Japan.2_2022
LC790059	Japan.1_2022
LC771966	Japan.1_2019
LC175468	Japan.1_2016
LC597133	Indonesia.1_2016
LC769702	India.1_2021
LC769683	India.14_2019
LC769684	India.13_2019
LC769685	India.12_2019
LC769688	India.11_2019
LC769691	India.10_2019
LC769692	India.9_2019
LC769693	India.8_2019
LC769694	India.7_2019
LC769695	India.6_2019
LC769696	India.5_2019
LC769697	India.4_2019
LC769698	India.3_2019
LC769699	India.2_2019
LC769714	India.1_2019
LC769707	India.2_2018
LC769708	India.1_2018
LC769705	India.1_2017
OL336385	China.22_2021
OL336388	China.21_2021
OL336389	China.20_2021
OP037976	China.19_2021
OP037977	China.18_2021
OP037978	China.17_2021
OP037979	China.16_2021
OP037980	China.15_2021
OP037981	China.14_2021
OP037982	China.13_2021
OP037983	China.12_2021
OQ940072	China.11_2021
OQ940073	China.10_2021
OQ940074	China.9_2021
OQ940075	China.8_2021
OQ940076	China.7_2021
OQ940077	China.6_2021
OQ940078	China.5_2021
OQ940079	China.4_2021
OQ940080	China.3_2021
OQ940081	China.2_2021
OQ940082	China.1_2021
OL336383	China.6_2020
OL336384	China.5_2020
OL336387	China.4_2020
OQ940069	China.3_2020
OQ940070	China.2_2020
OQ940071	China.1_2020
OL336382	China.1_2019
MN996297	China.2_2018

MN996299	China.1_2018
MH922876	Canada.2_2018
MW661258	Canada.2_2019
MW661259	Canada.1_2019
MW661256	Canada.1_2018
KY905335	Australia.1_2016
